# A Complete Energy Model for Graphene Flake Growth with the Fewest Possible Dangling Bonds

**DOI:** 10.3390/nano15100723

**Published:** 2025-05-11

**Authors:** Ivan G. Grozev, Dobromir A. Kalchevski, Dimitar V. Trifonov, Stefan K. Kolev, Hristiyan A. Aleksandrov, Valentin N. Popov, Teodor I. Milenov

**Affiliations:** 1 “Acad. E. Djakov” Institute of Electronics, Bulgarian Academy of Sciences, 72 Tsarigradsko Chaussee Blvd., 1784 Sofia, Bulgaria; igrozev@gmail.com (I.G.G.); dobromirak@ie.bas.bg (D.A.K.); dtrifonov@ie.bas.bg (D.V.T.); skkolev@ie.bas.bg (S.K.K.); 2 Faculty of Chemistry and Pharmacy, Sofia University “St. Kliment Ohridski”, 1 J. Bourchier Blvd., 1164 Sofia, Bulgaria; haa@chem.uni-sofia.bg; 3 Faculty of Physics, Sofia University “St. Kliment Ohridski”, 5 J. Bourchier Blvd., 1164 Sofia, Bulgaria

**Keywords:** graphene flakes, graphene nanoclusters, chemical graph theory, minimal energy model, graphene nanoflakes with the fewest possible dangling bonds, growth

## Abstract

This work presents a complete energy model for graphene flakes’ growth with the fewest possible dangling bonds. The model is based on a simple equation that describes the binding energy of graphene flakes consisting of up to 10,000 carbon atoms. Moreover, we demonstrate that the model can accurately calculate the binding energy of a topologically and geometrically diverse array of graphene flakes. According to our calculations, the model can predict the binding energy of a graphene flake with a deviation error of about 2–3%. Hence, we envision that the complete energy model for graphene flakes presented here could be utilized as a novel alternative to conventional Monte Carlo simulation methods used to study graphene growth.

## 1. Introduction

Graphene flakes (GFs) are finite molecular fragments of an otherwise infinite graphene sheet. Since the discovery of graphene [1], perfect high-symmetry GFs have become attractive model systems for computational and pure theoretical studies. However, among perfectly high-symmetry GFs, those with a hexagonal shape are most prominent among Cu substrate-grown GFs, especially when using bottom-up approaches like CVD [2,3,4]. The regular hexagonal shape has been theoretically proven as a global energy minimizer for 2D graphene-based structures using Lennard-Jones-like potentials [5,6]. Furthermore, two noteworthy theoretical results can be found in the works of Harary and Harbort [7] and Fulep and Sieben [8], which are of key importance to the present work. Using combinatorics and graph theory, they have proven that building a polyhex structure by adding hexagons in a spiral way, one hexagon after another, ensures the minimal possible edge perimeter and site (vertex) perimeter for a given number of cells. From a physics point of view, a minimal possible perimeter structure could be assumed as equivalent to the structure with the minimal formation energy, i.e., the most stable structure. In the case of GFs, this should correspond to the fewest possible dangling bonds. However, a free (non-passivated) graphene edge undergoes the so-called edge reconstruction to overcome the valence deficit in edge atoms [9]. This leads to an overall lowering of the system’s energy. It is widely accepted that among the two most abundant graphene edge configurations (armchair and zig-zag configurations), the armchair configuration has a lower energy per carbon atom and thus is more stable. The local geometry of the armchair configuration edges allows for the formation of a triple bond between two adjacent carbon (C) atoms with dangling bonds [9]. However, it has been proven experimentally that GFs interacting with a metal substrate are most stable when structured as perfect hexagons with full zig-zag edges [10]. Moreover, using CVD, especially with almost perfect epitaxial matches like on Cu (111) surfaces, one can grow macroscopic single-crystal graphene with atomically smooth, full zig-zag edges and perfect hexagonal shape [11,12]. In contrast, using a plasma torch to produce free GFs results in most GFs having a circular (dodecagonal) shape with mostly armchair edges [13]. It is also known that even for GFs with minimal perimeters, the number of possible isomer structures grows exponentially [14,15]. Thus, an exhaustive search approach for all structurally stable GFs is not realistically feasible, especially for large-scale GFs, even in theory. The lack of uniqueness, which follows from the exponential growth of the number of structures, naturally extends to all potentially possible growth paths. The usual approach to such types of problems is to obtain the so-called lower and upper bounds of the quantity we are interested in. In our case, this is the GF’s perimeter, as an indicator directly corresponding to the number of dangling bonds. Nevertheless, the advantages of determining the most stable GF structures are evident. The overall thermodynamic stability is directly correlated to thermal and chemical stability, which are fundamental to all bottom-up formation processes and related process parameters such as reagent flow, pressure, growth speed, and nucleation time. Moreover, important physical properties of graphene, such as band gap opening, are known to be sensitive to size and shape [16]. For example, hexagonal flakes have the most prominent metallic properties among the various GF topologies.

The model is based on a multidisciplinary approach, combining techniques and results from combinatorics/graph theory, appended with quantum chemical calculations. A schematic representation of the general idea and its realization in the final model is shown in Figure S1 in the Supplementary Materials. Using this approach, we derive a formal relationship, showing in an explicit form the dependence of the GF energy on its structural characteristics. It is shown that the model predicts the GF energy with a relative deviation of about 2–3%, most likely maintaining this accuracy for clusters with up to about 10,000 atoms.

Our original motivation for this study was to determine the construction of graphene clusters with the fewest possible dangling bonds for a given size. It is known that such configurations are ground states for graphene nanoflakes based on purely mathematical reasoning [5,6]. However, we found that the idea and methodology can be applied not only to the description of ground-state configurations but also to most morphological forms of graphene. The initial idea of accurately quantifying the edges of graphene clusters in terms of the type and number of bonds was extended to a comprehensive qualitative and quantitative description of entire clusters. The concept of representing the formation energy of compounds as a sum of their constituent bonds is longstanding and can be traced back to Herndon (1974) [17]. We found two works on graphene flakes that are relevant to our study. In the first one, Hendra and Witek [18] correlated various geometrical features (length, width, size, etc.) of rectangular graphene flakes with calculated density functional tight-binding total energies.

In the second paper, Fthenakis [19] focused on the study of GFs with a perfect hexagonal shape and provided an analytical expression for formation/cohesive energy as a function of size, which makes it closer to our work than the first one. The problem of studying only the perfect hexagonal shape is that there are many possible structures between two consecutive perfect flakes.

As discussed previously, the GFs with the fewest possible dangling bonds are the most stable structures, i.e., the ones with minimal formation energy [5,6,7]. In general, the formation energy of a compound can be represented as the sum of the constituent bond energies [17].

## 2. Theoretical Model

**Figure 1:**
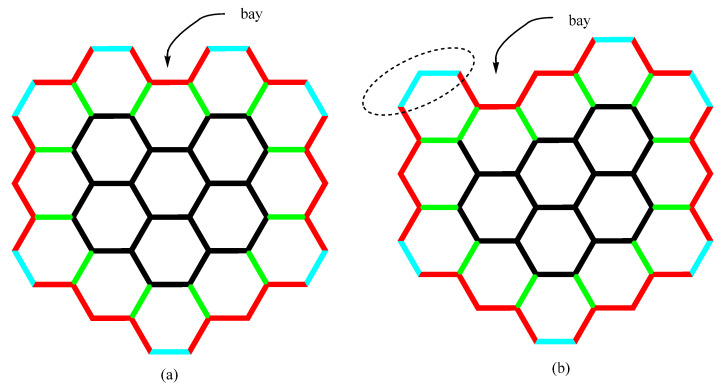
Schematic of the types of C-C bonds in two distinctive graphene flakes, represented as colored graphs; black edges: (-sp_c_^2^-sp_c_^2^-)-bonds, green edges: (-sp_c_^2^-sp_e_^2^-)-bonds, red edges: (-sp_e_^2^-sp_e_^1^-) and (-sp_e_^2^-sp_e_^2^-), cyan edges: (-sp_e_^1^-sp_e_^1^-). (**a**) Without (-sp_e_^1^-sp_e_^1^-sp_e_^1^-) segment and (**b**) with (-sp_e_^1^-sp_e_^1^-sp_e_^1^-) segment, enclosed in dashed ellipse.

The model presented here is based on the idea that the formation energy (binding energy) *E*_bind_ of a GF *H_m_* is the sum of the energy contributions of all the bonds in the flake. Hence, to calculate the formation energy of a given GF *H_m_*, we need to classify and enumerate the chemical bonds in *H_m_* correctly. The formation energy can generally be represented as

(1) $$E\left(H_{m}\right)=\sum_{i,j}\varepsilon_{ij},$$

where *i* and *j* denote the summation of the types and numbers of bonds, respectively. In our case, the bonds are classified into 4 types. A graphical presentation of all types of bonds is given in Figure 1.

Three of these types have a straightforward chemical and graph theory basis. The fourth classification type comes as a robust conclusion only after the analysis of optimized geometries. Our assumptions are supported in this respect by the work of A. V. Vorontsov and E. V. Tretyakov [20], both through semiempirical PM7 and DFT calculations. All 4 classification types are based on bond length values as the most important factor for determining bond strength. The numbering of every type is explicit and exact and is based on combining results from graph theory on planar hexagonal graphs.

Some useful definitions of the main physical characteristics of GFs and their graph theory correspondence are listed in Table 1.

With V’_3_, we denote deg(3) vertices that have at least one connection with V_2_; with V”_3_, we denote such vertices that have only connections with V_3_. With these definitions and the ones from Table 1, we make the following correspondences between the graph vertices and the GF atoms’ hybridization state:

V”_3_ → sp_c_^2^, V_2_ → sp_e_^1^, V’_3_ → sp_e_^2^.

The colors encoded in Figure 1 are as follows: blue edges, sp_e_^1^-sp_e_^1^ bonds; red edges, sp_e_^1^-sp_e_^2^ bonds plus sp_e_^2^-sp_e_^2^ (in the bay); green edges, sp_e_^2^-sp_c_^2^; black edges, sp_c_^2^-sp_c_^2^; and *e* and *c* subscripts denote flake boundaries (edges) and cores.

The boundaries are defined as the union of all bonds and atoms that form the following types of C-C bonds sp_e_^1^-sp_e_^1^, sp_e_^1^-sp_e_^2^, and sp_e_^2^-sp_e_^2^ (bay). Transition bonds (zone) are defined as the union of {all C-C bonds sp_e_^2^-sp_c_^2^}, and the GF core is defined as all atoms and bonds that form sp_c_^2^-sp_c_^2^ types of interaction.

As shown in Figure 1b, when a single boundary cell shares only two edges, there are 3 consecutive equivalent C atoms with dangling bonds: (-sp_e_^1^-sp_e_^1^-sp_e_^1^-). From a chemical point of view, only two options regarding chemical bonding in this segment are possible. Evidently, regarding bond type, only one triple bond can form in this segment. So, the two possibilities are (-sp_e_^1^≡sp_e_^1^-sp_e_^2^-) and (-sp_e_^2^=sp_e_^2^=sp_e_^2^-). The analysis of the bond lengths in the studied structures shows that the first scenario can be found more often; hence, we adopt it in our theoretical model. Flakes with such a structural motive are observed whenever a new wall of the flake starts to form.

**Figure 2:**
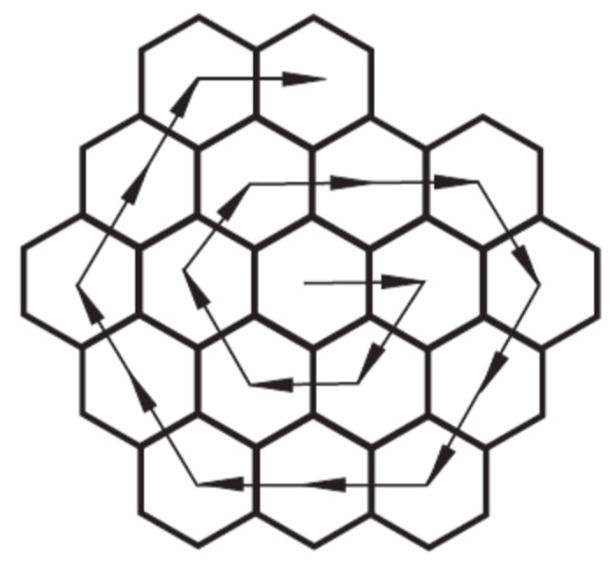
The Harary–Harborth construction of hexagonal systems with the fewest possible external vertices and edges. The system is constructed by adding hexagons one by one along the indicated spiral line [7,22].

For the flake structure generation, we use the spiral construction by Harary and Harborth [7], shown in Figure 2.

According to the same authors, this construction ensures the smallest possible perimeter for a specific number of cells. However, it should be noted that it is not the only possible construction.

For bond enumeration, we use the following relations:

(2) $$P_{e}\left(n\right)=2\left\lceil \sqrt{12n-3}\right\rceil$$

—Harary and Harborth [7]

(3) $$P_{v}\left(n\right)=\left\lceil \sqrt{12n-3}\right\rceil +3$$

—Fülep and Sieben [8]

(4) $$v\left(n\right)=2n+1+\left\lceil \sqrt{12n-3}\right\rceil$$

—Harary and Harborth [7]

(5) n−e+v=1

where $\left\lceil \sqrt{12n-3}\right\rceil$ is a ceiling function with the argument $\sqrt{12n-3}$; *P_e_*(*n*) is the edge perimeter and corresponds to the total number of bonds at the boundary (edge) of the flake; *P_v_*(*n*), the site perimeter, is equal to the number of carbon atoms with a valence deficit (dangling bonds); *v*(*n*) is the number of vertices, i.e., the number of all carbon atoms; *e* is the number of edges (bonds); and *n* is the number of cells (hexagons).

Then, based on Equations (2)–(5) and taking into account that there are 4 types of bond classification, we can rewrite Equation (1) as follows:

(6) $$E(n)=\alpha x\left(n\right)+\beta y(n)+\gamma z(n)+\delta t(n),$$

where *x*(*n*) is the number of (-sp_e_^1^-sp_e_^1^-) triple bonds, *y*(*n*) is the number of (-sp_e_^1^-sp_e_^2^-) bonds, *z*(*n*) is the number of (-sp_e_^2^-sp_c_^2^-) bonds, and *t*(*n*) is the number of (-sp_c_^2^-sp_c_^2^-) bonds; the coefficients *α*, *β*, *γ*, and *δ* denote the corresponding bond energy weight (measure).

The number of triple bonds (-sp_e_^1^≡sp_e_^1^-) for hexagonal topologies is at least 6. It can be seen (in Figure 1 and Figure 2) that the number of triple bonds for a hexagonal flake (generated by spiral construction) is either 6 or 7, depending on the flake wall completion. If the flake has completed walls, then *x* = 6, since *x* = 6 + *b*, while *b* = 0. If the current wall is not completely built, i.e., flake has a bay *x* = 7, since *x* = 6 + *b*, while *b* = 1 [21,22]. The only exceptions from the abovementioned cases appear when a new wall starts being built, i.e., in the case when the first hexagonal cell connects to the already fully built zig-zag edge (Figure 1b). Here, it is accepted that *b* = 0, despite the presence of one bay. Figure 1 shows flakes with one bay (*b* = 1), *x* = 6 + *b*. For spiral construction, the possible values of *b* are 0 or 1, depending on the geometry of the flakes. Generally, in spiral construction, the parameter *b* can be expressed as a function of n, thus allowing a fully automatic generation. However, this is formally a bit tedious and beyond our research scope.

The total number of bonds in a graphene boundary is *P_e_*(*n*), so the number of (-sp_e_^1^-sp_e_^2^-) bonds is *y*(*n*) = *P_e_*(*n*) − *x*. On the other hand, it is clear from Figure 1 that there is a one-to-one correspondence between *z*(*n*) and the number of sp_e_^2^ atoms. So, *z*(*n*) is the difference between the total number of edge atoms and the atoms with dangling bonds: *z*(*n*) = *P_e_*(*n*) − *P_v_*(*n*). Equation (5) is the Euler characteristic for a planar graph. Finally, for core bond numbers *t*(*n*), we have *t*(*n*) = total number of bonds − (*x* + *y* + *z*). Substituting *e* from Equation (5) into *t*(*n*) as well as *x*(*n*), *y*(*n*), and *z*(*n*) in *t*(*n*) yields *t*(*n*) = *n* + *v* − 1 − 2*P_e_*(*n*) + *P_v_*(*n*). Finally, considering *P_e_*(*n*), *P_v_*(*n*), *e*, and *v* from Equations (2)–(5), we obtain the following for *x*(*n*), *y*(*n*), *z*(*n*), and *t*(*n*):

(7) $$x\left(n\right)=6+b$$

(8) $$y\left(n\right)=2\left\lceil \sqrt{12n-3}\right\rceil -b-6$$

(9) $$z\left(n\right)=\left\lceil \sqrt{12n-3}\right\rceil -3$$

(10) $$t\left(n\right)=3n-2\left\lceil \sqrt{12n-3}\right\rceil +3$$

Substituting *x*(*n*), *y*(*n*), *z*(*n*), and *t*(*n*) from Equations (7)–(10) into Equation (6), and after performing some algebraic transformations, we obtain

(11) $$E\left(n\right)=3\delta n+\left(2\beta +\gamma -2\delta \right)\left\lceil \sqrt{12n-3}\right\rceil +\left(\alpha -\beta \right)\left(b+6\right)+3\left(\delta -\gamma \right)$$

Equation (11) is the main result of our model. The coefficients α, β, γ, and δ can be determined by an appropriate fitting procedure applied to a pre-calculated set of GF energies. For the latter, the PM6 method was used. The sum of the squares of the differences was chosen as the minimization criterion in the fitting procedure {*E*^model^ − *E*^PM6^}. It should be noted that Equation (11) is a nonlinear and non-smooth function. This fact requires the special selection of the methodology for the fitting procedure. The Generalized Reduced Gradient (GRG) and Evolutionary methods were used in this case. The GRG method cannot guarantee that the calculated solution is a global minimum due to the mentioned features of Equation (11). On the other hand, the Evolutionary method is suitable for non-smooth functions, which is much more time-consuming and, due to its nature, does not guarantee that the resulting solution is a global minimum. For these reasons, we use the following strategy: for initial fitting, the GRG method was used until an optimal solution was found; then, in several successive steps, we used the Evolutionary method until we obtained a stable (unchanging) solution. Finally, the following values are obtained for the coefficients in Equation (11)

*α* = 0.072491, *β* = 0.131265, *γ* = −0.142432, and *δ* = 0.003739 [hartree]

Now we can rewrite Equation (11) in a more useful form by replacing the coefficients with their numerical values and converting them to eV units of energy to obtain the following:

(12) $$E\left(n\right)=0.305222n+3.064552\left\lceil \sqrt{12n-3}\right\rceil -1.599304b+2.336677\;[\mathrm{eV}]$$

A natural way of obtaining the four unknown coefficients {*α*, *β*, *γ*, *δ*} is to construct and solve a system of four equations in the form of Equation (6), as follows:

(13) $$\left|\begin{array}{c} \alpha x\left(n_{1}\right)+\beta y(n_{1})+\gamma z(n_{1})+\delta t(n_{1})=E^{PM6}(n_{1})\\ \alpha x\left(n_{2}\right)+\beta y(n_{2})+\gamma z(n_{2})+\delta t(n_{2})=E^{PM6}(n_{2})\\ \alpha x\left(n_{3}\right)+\beta y(n_{3})+\gamma z(n_{3})+\delta t(n_{3})=E^{PM6}(n_{3})\\ \alpha x\left(n_{4}\right)+\beta y(n_{4})+\gamma z(n_{4})+\delta t(n_{4})=E^{PM6}(n_{4}) \end{array}\right.$$

By solving Equation (13) using a precise selection of flakes {*H_n_*_1_, *H_n_*_2_, *H_n_*_3_, *H_n_*_4_}, one can obtain results that are very close to the predictions of Equation (12). For example, using the information about the clusters {C_59_, C_99_, C_144_, C_157_}, a set of values of the coefficients {*α*, *β*, *γ*, *δ*} is derived, leading to a variant of Equation (12) with good qualitative and quantitative correspondence of the predicted energy values. The example provided above is not an isolated case. As shown in Table S1 in the Supplementary Materials, most flake predictions of Equation (12) fall within a 2% error margin from the PM6 energy, which is comparable to the results of different quantum computational methods. Based on the value of the coefficients {*α*, *β*, *γ*, *δ*}, their direct interpretation as corresponding bond strengths does not seem to be reasonable. By definition, if *α*, *β*, *γ*, and *δ* represent the bond energies of, respectively, *α*: (sp_e_^1^-sp_e_^1^) (implying a bond order of 3); *β*: (sp_e_^1^-sp_e_^2^)ꓴ(sp_e_^2^-sp_e_^2^), which has an order between single and double bonds but is closer to a double bond; *γ*: (sp_e_^2^-sp_c_^2^), which has a bond order between 1 and 2 but closer to 1; and *δ*: (sp_c_^2^-sp_c_^2^), which represents a delocalized bond with an order between a double and single bond, one might thus expect that the values of the coefficients would be arranged as follows, *α* ˃ *β* ≥ *δ* ˃ *γ*, but instead, the order is *β* ˃ *α* ˃ *δ* ˃ *γ*. First, we must consider that all flake formation energies are positive, resulting from the fact that in PM6, only valence electrons are considered, similar to all semiempirical methods. Therefore, the absolute value of the PM6 energy is expected to be much larger than that of non-pseudopotential methods like ab initio. Secondly, in the PMx methods, the parametrization for carbon yields a slight positive formation energy for graphite, which has a standard enthalpy of formation Δ*H_f_* = 0 by definition [23]. With this parametrization, the formation energy of all flakes should be positive, as they must possess higher formation energy than “infinite” graphite. The value of *δ* is very close to zero but remains positive. This is reasonable, as this coefficient represents bonds in the core of the flake, similar to those in ideal graphene. The negative value of *γ* may seem unusual, as we expect it to have the highest energy due to the longest length of this type of bond. However, as indicated in Equation (11), its functional dependence is not straightforward and does not change the sign of the second term. Also, in the last term of Equation (11), there is a negative sign in front of *γ*, so its effect on this term is to raise the energy, as expected. The third term in Equation (11) describes the impact of triple bonds due to the (*α* − *β*) part, and if *α* ˃ *β*, this term will increase the energy, which would be physically incorrect; hence, *α* < *β*. Moreover, the result *β* ˃ *α* is extremely significant. Examining Equation (12), it becomes clear that because *β* ˃ *α*, the third term in the equation is negative. It should be noted that this is the only negative term, leading to a decrease in energy. In other words, *β* ˃ *α* implies that the formation of a triple bond (carbyne) along the GF boundary, due to boundary reconstruction, results in a greater energy reduction than the double (carbene) bond formation. In summary, the functional dependence described by Equation (11) is complex and requires further analysis.

## 3. Computational Method, Results, and Details

Here, a total of 121 graphene flakes were studied. A full list of all cluster energies predicted by the model, PM6 energy, and structural characteristics is given in the Supplementary Materials. The smallest cluster is C_24_, and the largest is C_294_. Each structure was generated by adding one hexagon to the previous one (Figure 2). The initial geometry of all clusters was chosen to be the same as that of perfect graphene, with all bonds set to 1.42 Å and all angles set to 120°. Geometry optimization was performed using the PM6 semiempirical method within the Gaussian09 software package [24]. A singlet spin state was assumed for all clusters. Since the investigated effect is expected to be mostly structural and geometric, we employed the PM6 method as it provides reliable geometry for graphene structures [25,26]. After geometry optimization, if a global minimum of energy was not found, it was performed again with a slightly changed initial geometry. This procedure was repeated until a global energy minimum was found. The energy set {E_24_, E_27_, E_30_, …, E_292_, E_294_} represents the most important data from the PM6 calculations, as we directly used it to build our theoretical model.

**Figure 3:**
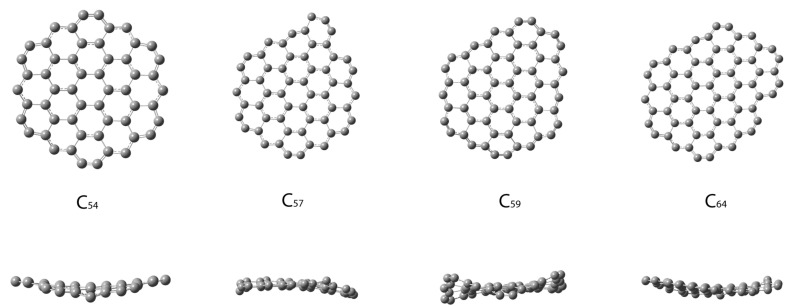
Front (**up**) and side (**down**) views of some morphologically representative graphene flakes after geometry optimization.

Examples of optimized geometries of the GFs C_54_, C_57_, C_59_, and C_64_ (front and side views) are given in Figure 3.

A significant edge reconstruction is observed in all flakes. In general, the bond length contraction appears in almost all edge bonds. The type 4 bond classification adopted in the model agrees well with the computational results.

**Figure 4:**
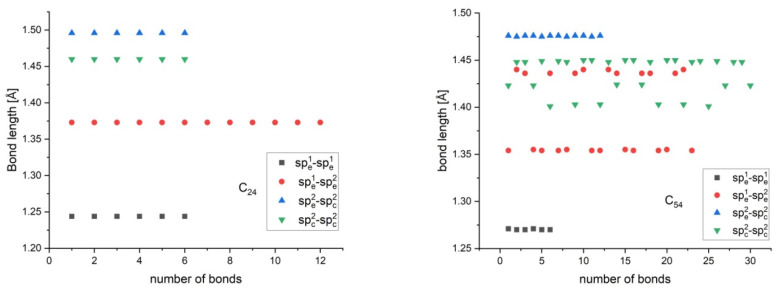

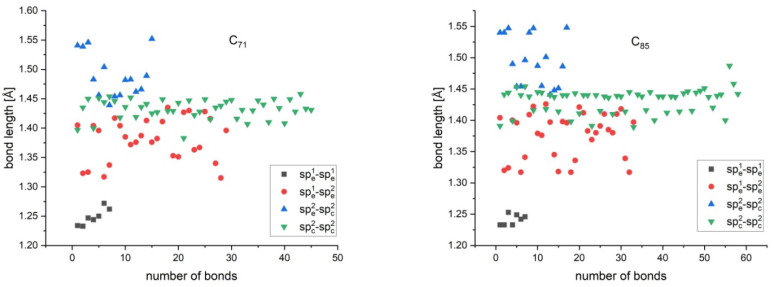
Type 4 bond length distribution for clusters C_24_, C_54_, C_71_, and C_85_.

In addition, the bond length analysis of most graphene flakes also validated our assumption for 4 bond types. Normally, the spiral construction as a building algorithm starts at C_6_. However, we decided to start with a larger structure, namely C_24_. The reason for this is that the properties of the small flakes are expected to deviate significantly from those of the larger flakes. This is also evident from our results. In the case of C_24_, the predicted energy has the largest deviation, even though it possesses a perfect bond length separation (Figure 4). Additionally, C_24_ is the smallest flake in which the 4-bond type classification is reasonable. Moreover, it is known that, for some small flakes like C_14_, C_18_, and C_22_, the polyhex form is unstable, and after geometry optimization, it reorganizes into a large monocyclic structure [27].

The bond length distribution for some clusters is presented in Figure 4.

**Figure 5:**
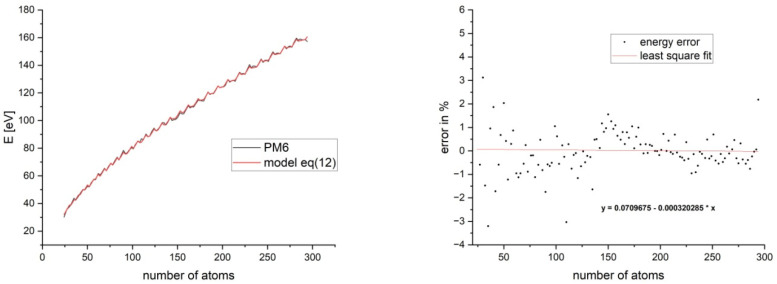
GF energies from PM6 calculations and energies predicted by Equation (12) as a function of the number of C atoms (**left**) and error distribution (%) as a function of size, together with the equation of the linear least square fit of error distribution (**right**).

As we already mentioned, separating the sp_e_^2^-sp_c_^2^ C-C bonds as a distinct bond type is a result of the analysis of the bond length distribution. Indeed, from Figure 4, a relatively good separation of the {sp_e_^2^-sp_c_^2^} bonds from the remaining bond types can be observed. This is most obvious for highly symmetric flakes like C_24_, C_54_, and C_96_ that possess D_6h_ symmetry. The existence of this “transition bonds” region can be explained by the fact that the flake core is a network of three coordinated carbon atoms, all in the sp^2^ hybridization state with no valence deficits. The lowest energy state is the one with the optimal delocalization of π-electrons, and this is possible only if the flake core is flat. On the other hand, the edge region contains unsaturated C atoms and can be stabilized via reconstructions. Thus, the core has a distorted, out-of-plane structure. Hence, the flake core and edge have two mutually opposing preferred optimal geometric states. An overall energy minimum will be reached if these two regions are separated as much as possible; hence, a transition zone (bonds) appears with longer bond lengths. Figure 4 shows a substantial overlap and scatter between the {sp_e_^1^-sp_e_^2^} and {sp_c_^2^-sp_c_^2^} bonds, especially for larger flakes. From here, one may assume that the 4-type classification is unnecessary and that a 3-type classification, which unifies the {sp_e_^1^-sp_e_^2^} and {sp_c_^2^-sp_c_^2^} bonds under one group, will be reasonable. This approach is erroneous as it contradicts the fundamental distinction between the boundary and core regions. This will lead to qualitative and quantitative differences, because Equation (11) will change significantly. Fthenakis [19] proposes a 3-type bond model (2 types for boundary and 1 for the core). We tested this 3-type bond model and found that it yields 1.5–2 times larger deviation from the PM6 energy deviations. Thus, a 4-type model is the optimal one concerning precision and simplicity. The energies *E*^PM6^ and *E*^model^ (see Equation (12)) are shown in Figure S2 (Supplementary Materials). Their qualitative and quantitative similarities are evident when plotted together (Figure 5).

From the slope of the error distribution in Figure 5 (right) and relying on the linear fit, we can conclude that the model can predict the energy of GFs with up to 10,000 atoms within a 2–3% deviation. A more detailed analysis regarding the extrapolation of the model for larger clusters can be found in the SMs. Equation (12) allows us to make a relative estimation of the binding energy for any GF size and shape, including GFs with topologies and geometries different from the ones we used to build the model. We also tested the reliability of the model on the following six structures: (a) C_108_ (rectangular flake), (b) C_144_ (hexagonal flake with internal 6C-atom defect), (c) C_282_ (dodecagonal flake), (d) C_108_ (one-side-elongated hexagonal flake), (e) C_123_ (elongated rhomboidal flake), and (f) C_114_ (armchair hexagonal flake).

**Figure 6:**
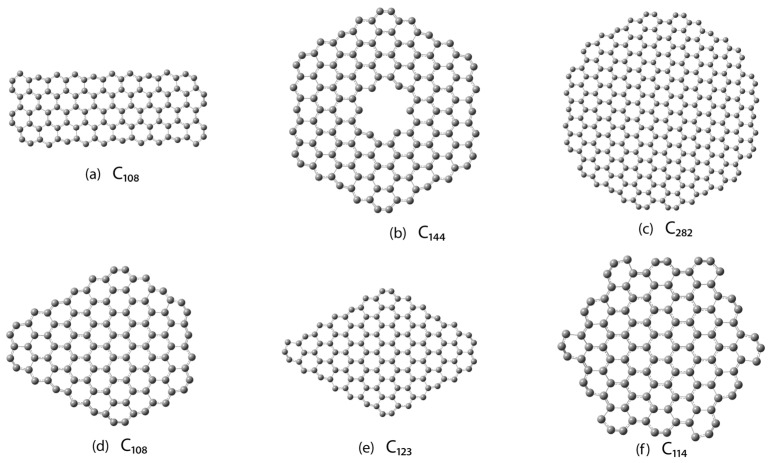
Example of six flakes used for cross-validation of the model.

Numerical values of energy as well as errors are given in Table 2. Each of the six flakes in Figure 6 represents unique morphological features. For example, flake (a) can be seen as a nanoribbon. Flake (b) represents a flake with an internal defect. Flakes (d) and (e) can be seen as hexagonal C_96_, on which, in the case of (d), only one of the sides has been grown with 2 layers of cells, and, in the case of (e), two sides have been grown. The significantly larger error in the case of C_144_ can be explained by the fact that the internal edges are much more different from the external ones. For example, no reconstruction in the internal edges leads to triple bonding. The overall reconstruction in this region is not as dominant as in the cases with external edges. This explains why the model predicts a lower energy of such flakes compared to their actual value (Table 2).

Flakes C_282_ and C_114_ are even more special. As mentioned previously, C_282_ is an example of the smallest flake with a satisfactory minimal perimeter (dangling bonds) and maximal bay (b) numbers, *b* = 6. According to our main equation, such a flake must represent the global energy minimum. Indeed, comparing the energy of a spirally constructed C_282_ to the one shown in Figure 6 (a dodecagonal flake), there is a significant energy difference of more than 15 eV. We compare the two structures in Figure S3 (Supplementary Materials). Moreover, the growth of such octagonal flakes is an isotropic process; i.e., it allows a simultaneous growth/etching on all six sides of the hexagonal flake. Graphene clusters with such a structure (1 < *b* ≤ 6) present a strong argument that a theoretical model of the growth mechanism of graphene clusters should contain such structures. Indeed, this grown pattern has been observed both experimentally [28] and theoretically in Monte Carlo simulations [29]. C_114_ is an example of a hexagonal flake with a fully armchair edge. As it has already become clear in the current model, which is a theory for minimal perimeter flakes, zig-zag edges are the dominant type of boundary. On the other hand, as one can see from Table 2, the current model predicts well the energy of a fully armchair flake. Yet, we believe that establishing an analogous theoretical model for armchair graphene flakes is of emerging importance for a deeper understanding of graphene flakes.

As mentioned above, we found two publications related to our research [18,19]. The former is for hydrogen-passivated flakes, while the latter allows for only a partial energy comparison to our results. Since the model in [19] is constructed solely for perfect hexagonal structures (C_24,_ C_54_, C_96_…), it is expected to provide more accurate estimation for such structures compared to our model. On the contrary, our model is about 6 to 7 times more accurate for structures different from perfect hexagons.

We will also highlight several important aspects of the model in terms of possibilities for potential applications: (i) The model can be a tool for the rough but fast estimation of the energy of a large number of cluster structures, both isomeric to the ones studied in this work and to those with a different topology. (ii) The model can be the basis for studying and constructing potential mechanisms of cluster growth near equilibrium conditions. (iii) The model can be used to establish the relationship between structural characteristics and physical properties of graphene-like nanoclusters. Geometry, topology, and size can be mathematically correlated to a band gap (hence excitation energy), frequencies of normal vibrational modes, etc. (iv) The model can be of interest as an algorithmic basis for machine learning in the field of graphene nanoclusters research.

Some parallel analogies can be made between our model and Monte Carlo approaches to graphene growth: (i) Both methods rely on elementary events: a single aromatic ring attachment, as in our case, and usually from a single C atom up to a single aromatic ring in the case of kinetic Monte Carlo. (ii) Energy parameters for elementary events come from independent methods, usually ab initio MD. (iii) Regarding graphene, in both approaches, the main task is to investigate the possible growth paths.

The potential improvement of the model can be achieved along the following lines: (i) The direct quantitative improvement of the predicted energies can be achieved by replacing the semiempirical PM6 method with more accurate ab initio methods. (ii) Conceptual improvements to the model can be based on the discovery of further bond decompositions of the edge and core of the cluster. (iii) Finally, we emphasize the importance of a model describing clusters with dominant armchair edges.

## 4. Conclusions

In this work, we presented a new model for calculating the energies of hexagonal graphene flakes. The flakes are generated continuously by adding one hexagonal cell at each step. This is equivalent to a growth pattern with two and occasionally three carbon atoms. So far as we know, this is the first energy model that allows simulating such a fine incremental growth besides accurately predicting the binding energy of flakes with various topologies from an original set of structures. Based on the idea that the binding energy can be represented as the sum of individual bond energies, the model is a unification of the explicit numbering of carefully classified bond types, and this classification is proven by both graph theory and quantum chemical methodology.

## Figures and Tables

**Table 1:** Correspondence between the physical constituent of a graphene flake and the graph theory analogy.

Graphene’s Flake Component	Graph Theory Definition
Graphene flake (GF)	Graph G(E,V)
Number of carbon atoms and hexagon rings in the GF Number of bonds in the GF	Number of vertices(V)≡|V| and number of hexagons(n)≡|n|
Number of bonds in the GF	Number of edges|≡|E|
Coordination number of carbon atoms: C(2), C(3)	Vertex degree, deg(V)≡V(deg):V_2_, V_3_
GF boundary	∂G(V,E)≡{V_2_}ꓴ{V’_3_}
GF core	Gc(V”_3_,E), Gc(V”_3_,E)⸦G(V,E)

**Table 2:** PM6 and model energies of six tested flakes with different morphologies.

Flake	PM6 [eV]	Model [eV]	Error [%]
C_108_	97.29	99.12	−1.85
C_144_	123.05	117.22	−4.74
C_282_	143.86	149.20	3.71
C_108_	85.75	85.64	−0.13
C_123_	98.12	98.27	−0.15
C_114_	87.38	88.61	1.41

## Data Availability

Data is contained within the article.

## Supplementary Materials

The following supporting information can be downloaded at https://www.mdpi.com/article/10.3390/nano15100723/s1: Table S1. Results of PM6 and predicted by the model energies of all graphene clusters. Figure S1. Calculated PM6 energy, *E^PM6^*, (up) and the energy predicted by Equation (12), *E^model^*, (down) as a function of the GF number of atoms. Figure S2. Graphene flakes C_282_ generated by spiral construction (up) and C_282_ with dodecagonal morphology (down). Figure S3. General scheme for establishing an energy growth model for periodic (crystalline) 2D materials. Figure S4. Energy error distribution with linear fit/equations for sub-models M_(24–117)_, M_(119–206),_ and M_(208–292)_.

## Abbreviations

The following abbreviations are used in this manuscript:

GF	Graphene flake
CVD	Chemical vapor deposition
GFs	Graphene flakes
DFT	Density functional theory
PM6	Parameterization Method 6
