# Supplementary material for: A Complete Energy Model for Graphene Flake Growth with the Fewest Possible Dangling Bonds
Source: Nanomaterials (Basel). 2025 May 11;15(10):723. doi: 10.3390/nano15100723 (PMC12113863; doi:10.3390/nano15100723)
Supplement: Supplementary file 1 [file nanomaterials-15-00723-s001.zip › nanomaterials-3587939-supplementary.pdf]

SUPLEMENTARY MATERIALS

Table S1. Results of PM6 and predicted by the model energies of all graphene clusters.

n	*C atoms*	*b*	*x(n)*	*y(n)*	*z(n)*	*t(n)*	E^{model} *[eV]*	E^{PM6} *[eV]*	*Error [%]* = $(E^{model}-E^{PM6})100/E^{PM6}$
7	24	0	6	12	6	6	32.05	30.22	6.06
8	27	0	6	14	7	7	35.42	35.63	−0.59
9	30	0	6	16	8	8	38.79	37.62	3.12
10	32	0	6	16	8	11	39.10	39.69	−1.48
11	35	0	6	18	9	12	42.47	43.88	−3.21
12	37	0	6	18	9	15	42.77	42.37	0.95
13	40	0	6	20	10	16	46.14	45.30	1.86
14	42	0	6	20	10	19	46.45	47.26	−1.72
15	45	0	6	22	11	20	49.82	50.11	−0.59
16	47	0	6	22	11	23	50.12	49.79	0.68
17	50	0	6	24	12	24	53.49	52.43	2.03
18	52	1	7	23	12	27	52.20	51.98	0.42
19	54	0	6	24	12	30	54.10	54.78	−1.22
20	57	0	6	26	13	31	57.47	57.30	0.30
21	59	0	6	26	13	34	57.78	57.28	0.87
22	62	0	6	28	14	35	61.15	61.74	−0.96
23	64	1	7	27	14	38	59.86	60.58	−1.13

24	66	0	6	28	14	41	61.76	62.35	−0.95
25	69	0	6	30	15	42	65.13	65.49	−0.55
26	71	1	7	29	15	45	63.84	63.68	0.24
27	73	0	6	30	15	48	65.74	66.33	−0.89
28	76	0	6	32	16	49	69.11	69.25	−0.20
29	78	1	7	31	16	52	67.82	67.95	−0.19
30	80	0	6	32	16	55	69.72	70.51	−1.12
31	83	0	6	34	17	56	73.09	73.53	−0.60
32	85	1	7	33	17	59	71.80	71.46	0.47
33	87	0	6	34	17	62	73.70	74.31	−0.82
34	90	0	6	36	18	63	77.07	78.44	−1.75
35	92	1	7	35	18	66	75.78	76.22	−0.58
36	94	1	7	35	18	69	76.08	76.57	−0.64
37	96	0	6	36	18	72	77.99	78.38	−0.51
38	99	0	6	38	19	73	81.36	80.51	1.05
39	101	1	7	37	19	76	80.06	79.57	0.62
40	103	0	6	38	19	79	81.97	82.42	−0.55
41	106	0	6	40	20	80	85.34	85.14	0.23
42	108	1	7	39	20	83	84.04	84.26	−0.25
43	110	1	7	39	20	86	84.35	86.99	−3.03
44	112	0	6	40	20	89	86.25	86.01	0.28
45	115	0	6	42	21	90	89.62	90.31	−0.76
46	117	1	7	41	21	93	88.33	88.48	−0.18
47	119	1	7	41	21	96	88.63	88.72	−0.10

48	121	0	6	42	21	99	90.54	91.60	−1.16
49	124	0	6	44	22	100	93.91	94.53	−0.66
50	126	1	7	43	22	103	92.61	92.63	−0.02
51	128	1	7	43	22	106	92.92	93.37	−0.49
52	130	0	6	44	22	109	94.82	95.02	−0.21
53	133	0	6	46	23	110	98.19	98.45	−0.26
54	135	1	7	45	23	113	96.90	98.52	−1.64
55	137	1	7	45	23	116	97.20	96.75	0.47
56	139	0	6	46	23	119	99.11	98.62	0.50
57	142	0	6	48	24	120	102.48	102.35	0.12
58	144	1	7	47	24	123	101.18	100.01	1.17
59	146	1	7	47	24	126	101.49	100.67	0.81
60	148	1	7	47	24	129	101.79	100.82	0.96
61	150	0	6	48	24	132	103.70	102.11	1.55
62	153	0	6	50	25	133	107.07	105.74	1.26
63	155	1	7	49	25	136	105.77	104.79	0.94
64	157	1	7	49	25	139	106.08	104.93	1.09
65	159	0	6	50	25	142	107.98	107.30	0.64
66	162	0	6	52	26	143	111.35	110.82	0.48
67	164	1	7	51	26	146	110.06	109.19	0.80
68	166	1	7	51	26	149	110.36	110.05	0.29
69	168	1	7	51	26	152	110.67	109.80	0.79
70	170	0	6	52	26	155	112.57	111.95	0.56
71	173	0	6	54	27	156	115.94	114.75	1.04

72	175	1	7	53	27	159	114.65	114.53	0.11
73	177	1	7	53	27	162	114.96	114.27	0.60
74	179	1	7	53	27	165	115.26	114.13	0.99
75	181	0	6	54	27	168	117.16	116.84	0.27
76	184	0	6	56	28	169	120.53	120.67	−0.11
77	186	1	7	55	28	172	119.24	118.9	0.28
78	188	1	7	55	28	175	119.55	119.65	−0.09
79	190	1	7	55	28	178	119.85	119.55	0.26
80	192	0	6	56	28	181	121.76	121.5	0.21
81	195	0	6	58	29	182	125.13	125.13	−0.01
82	197	1	7	57	29	185	123.83	123.85	−0.01
83	199	1	7	57	29	188	124.14	124.36	−0.18
84	201	1	7	57	29	191	124.44	124.39	0.04
85	203	0	6	58	29	194	126.35	125.45	0.72
86	206	0	6	60	30	195	129.72	129.72	0
87	208	1	7	59	30	198	128.42	127.87	0.43
88	210	1	7	59	30	201	128.73	128.8	−0.06
89	212	1	7	59	30	204	129.03	129.2	−0.13
90	214	1	7	59	30	207	129.34	128.45	0.69
91	216	0	6	60	30	210	131.24	131.34	−0.08
92	219	0	6	62	31	211	134.61	134.94	−0.25
93	221	1	7	61	31	214	133.32	133.71	−0.29
94	223	1	7	61	31	217	133.62	134.16	−0.4
95	225	1	7	61	31	220	133.93	133.44	0.37

96	227	0	6	62	31	223	135.83	136.3	−0.34
97	230	0	6	64	32	224	139.20	140.55	−0.96
98	232	1	7	63	32	227	137.91	138.1	−0.14
99	234	1	7	63	32	230	138.21	139.48	−0.91
100	236	1	7	63	32	233	138.52	139.4	−0.63
101	238	1	7	63	32	236	138.82	138.88	−0.04
102	240	0	6	64	32	239	140.73	140.91	−0.13
103	243	0	6	66	33	240	144.10	144.54	−0.3
104	245	1	7	65	33	243	142.80	142.12	0.48
105	247	1	7	65	33	246	143.11	143.58	−0.33
106	249	1	7	65	33	249	143.41	143.74	−0.22
107	251	1	7	65	33	252	143.72	142.73	0.7
108	253	0	6	66	33	255	145.62	146.23	−0.41
109	256	0	6	68	34	256	148.99	149.81	−0.54
110	258	1	7	67	34	259	147.70	147.9	−0.14
111	260	1	7	67	34	262	148.01	148.71	−0.47
112	262	1	7	67	34	265	148.31	148.78	−0.32
113	264	1	7	67	34	268	148.62	148.35	0.18
114	266	0	6	68	34	271	150.52	150.69	−0.12
115	269	0	6	70	35	272	153.89	153.79	0.06
116	271	1	7	69	35	275	152.60	151.9	0.46
117	273	1	7	69	35	278	152.90	153.39	−0.32
118	275	1	7	69	35	281	153.21	154.02	−0.53
119	277	1	7	69	35	284	153.51	153.02	0.32

120	279	0	6	70	35	287	155.42	155.98	−0.36
121	282	0	6	72	36	288	158.79	159.66	−0.55
122	284	1	7	71	36	291	157.49	158.11	−0.39
123	286	1	7	71	36	294	157.80	159.01	−0.76
124	288	1	7	71	36	297	158.10	158.48	−0.24
125	290	1	7	71	36	300	158.41	158.46	−0.03
126	292	1	7	71	36	303	158.71	158.62	0.06
127	294	0	6	72	36	306	160.62	157.19	2.18

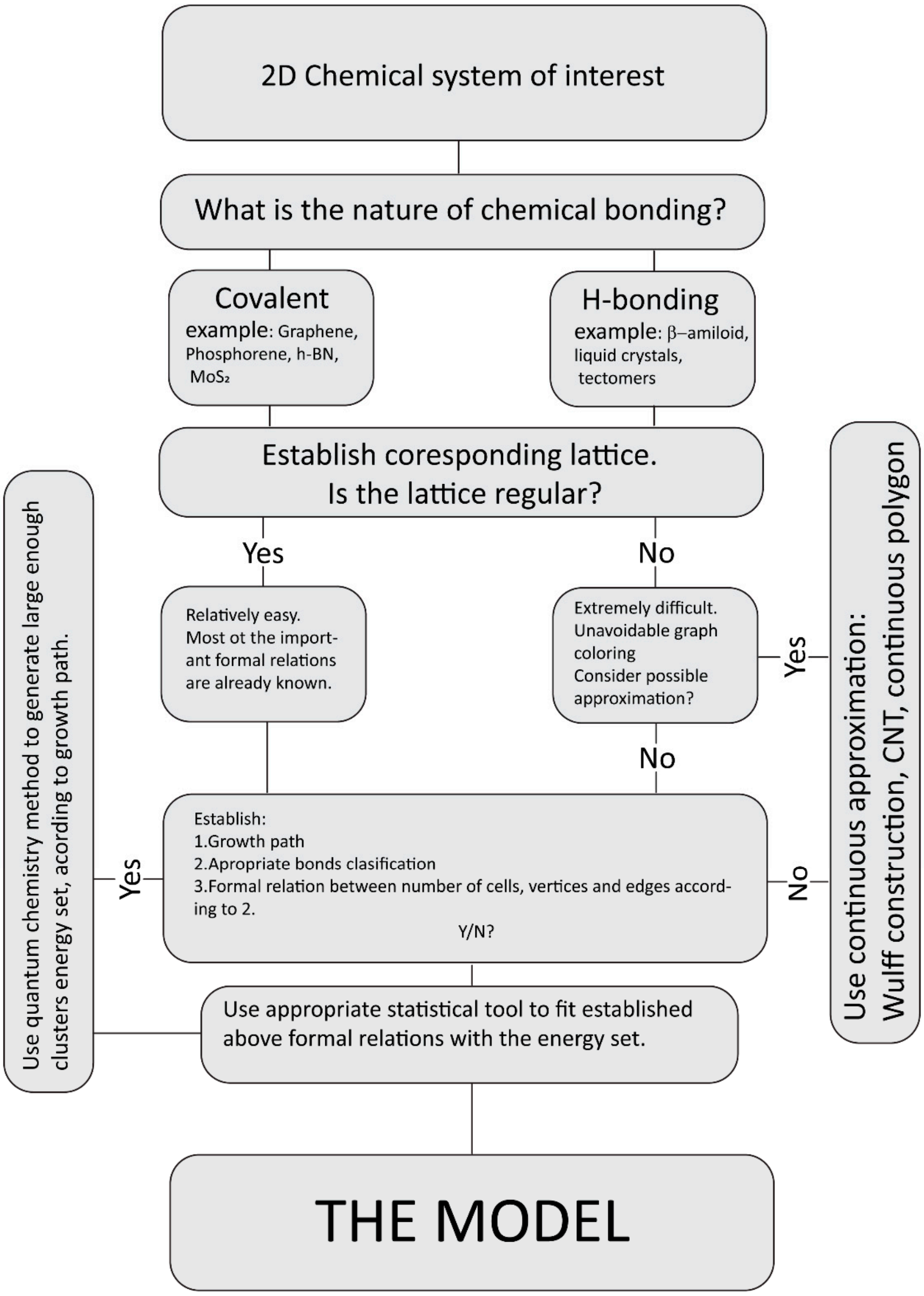

Figure S1. General scheme for establishing an energy growth model for periodic (crystalline) 2D materials.

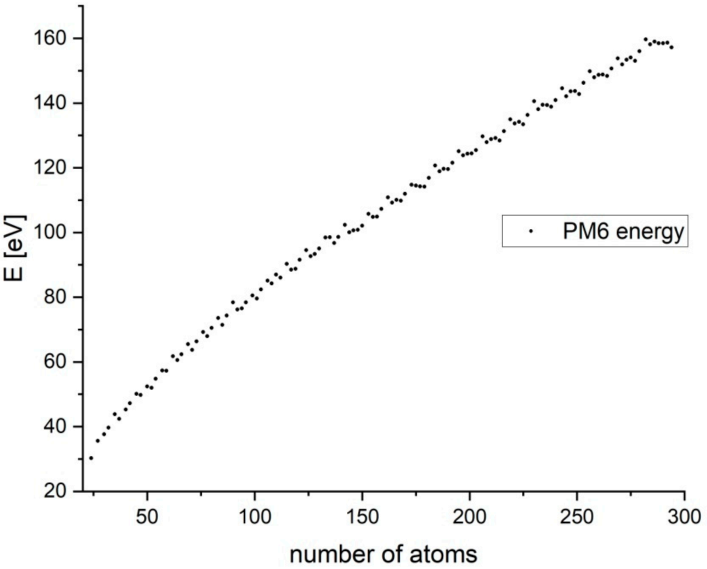

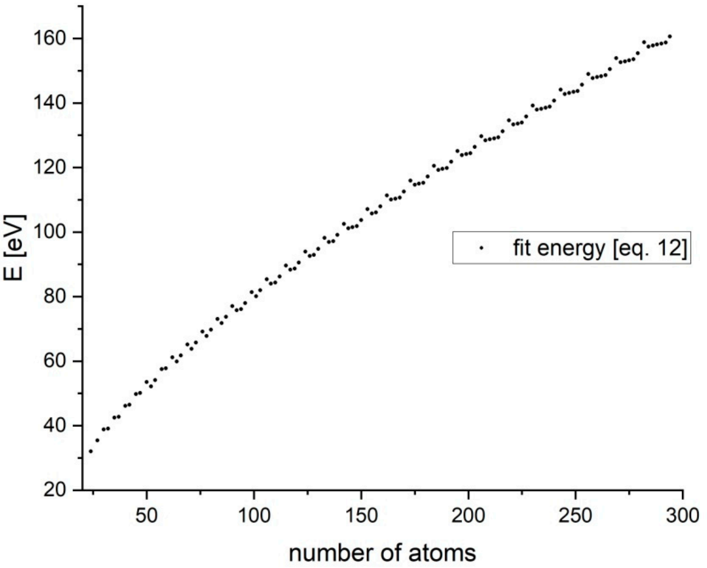

Figure S2. Calculated PM6 energy, E^{PM6}, (up) and the energy predicted by Eq. 12, E^{model}, (down) as a function of the GF number of atoms.

We emphasize on discrete energy growth evident on E^{PM6}. This shows that for any model to adequately describe the GF energy, it must be discrete. Our approach, using combinatorics/graph theory accomplishes this aim.

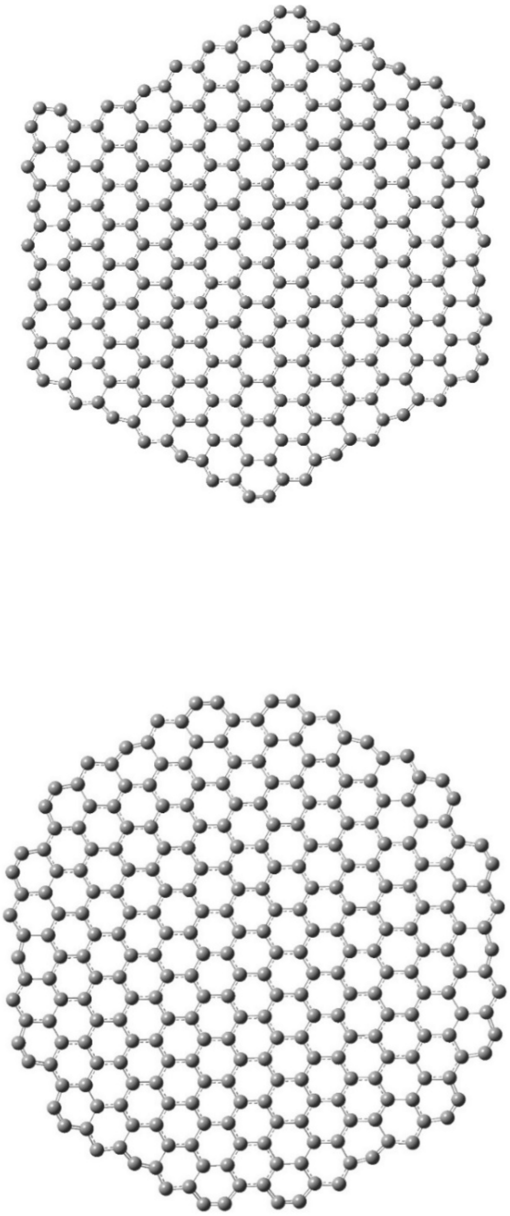

Figure S3. Graphene flakes C_{282} generated by spiral construction (up) and C_{282} with dodecagonal morphology (down).

To support our claim about the extrapolation of the observed trend, we present additional analysis. Within the model and known information, we can point out two major factors that can negatively influence the predictability of the model. These factors are incompleteness in the treatment of the edge region of the flake and incompleteness in the treatment of the flake's core. Now, as we can see from our results (Eq. 11), the edge scales approximately as $\sqrt{n}$. So any effect correlated with the edge is expected to diminish with the increase in the flake`s size. On the other hand, the core region scales as n and cannot be disregarded easily. Since we know that the core part of the flake is a delocalized honeycomb network of sp^2 hybridized C atoms, we expect the core flakes to be influenced by edge effects to some degree. In other words, parts of the core that are closer to the edge may be slightly different from parts of the core that are further away from the edge. A natural question that arises is how strong this effect is, and more importantly, how fast it diminishes (converges) to a constant value. In the limit $n \rightarrow \infty$, the bond energy in the core converges (probably asymptotically) to the bond energy of an infinite graphene sheet. One way to explore this convergence issue of the flake's core within our model is to analyze the slopes of the equations of error linear regression analysis of smaller independent models. We divided our energy set into three sets of equal size: {C_{24} – C_{117}}, {C_{119} – C_{206}}, and {C_{208} – C_{292}}. We denote the corresponding models as construct consecutive sub-models: $M_{(24\text{-}117)}$, $M_{\{119\text{-}206\}}$, and $M_{\{208\text{-}292\}}$. The same statistical analysis used for the full energy set now applies to each smaller set. The results are shown in the figures below.

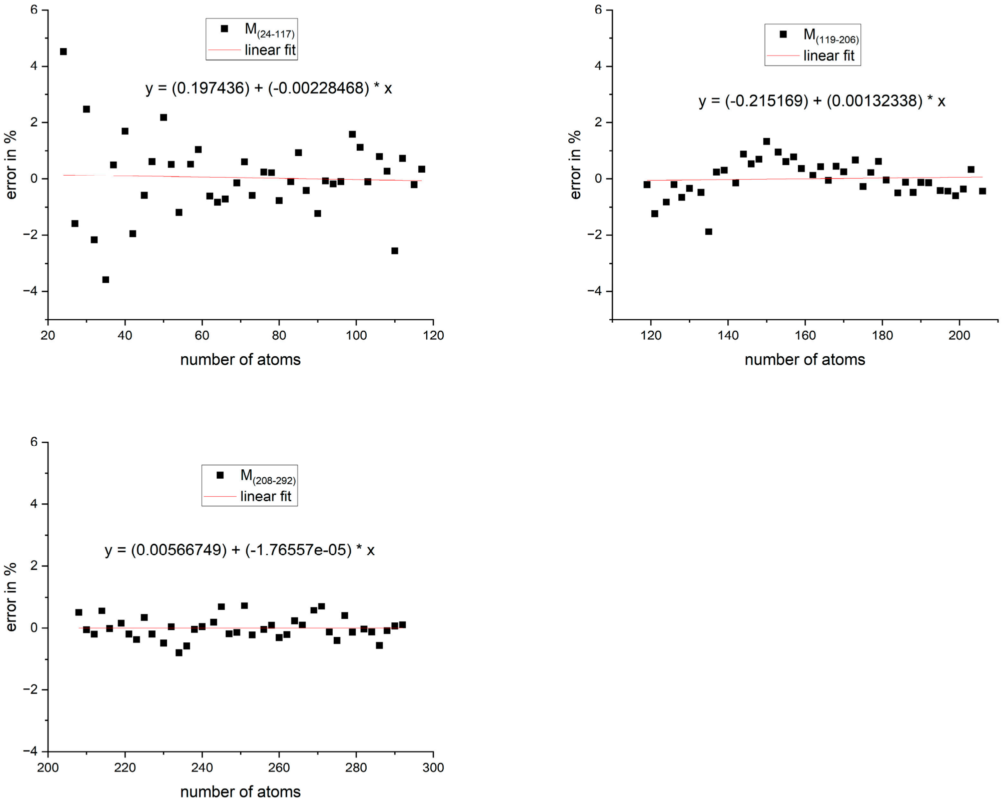

Figure S4. Energy error distribution with linear fit/equations for sub-models $M_{(24\text{-}117)}$, $M_{(119\text{-}206)}$, and $M_{(208\text{-}292)}$.

From the graphs in Fig. SM4, one can notice the following:

1. The slopes improve with shifting toward sub-models for bigger clusters.
2. The improvement is slightly shifting from $M_{(24-117)}$ to $M_{(119-206)}$ and 65 times shifting from $M_{(119-206)}$ to $M_{(208-292)}$.
3. All errors inside the fits and the verified ones (extrapolated) are smaller than the claimed 3%, and for bigger flakes are much below 3%.
4. The error appears to oscillate and to be bounded.

Taking into account the analysis that we made and the result above, we can conclude the following:

Convergence for the flake's core happens somewhere around C_{160}-C_{180}.

The error is unlikely to diverge with increasing the size, unless new effects appear.
